# 

**Published:** 1884-08

**Authors:** 


					﻿AUGUST, 1884.
tfLD SERIES
/ Vol. XXV
..& 1855 O
NEW SERIES
Vol. I.—No. 6.	|
WTHNK
HEDICMs^SRML
JOURNAL
-^7Q'Ma&ru^a.—i/Zt-dtA
GjtahJ
W.F. WESTMOREL AND, N®
h.v.m.miller,m.d.ll.dJ
JAMES A.GRAY, M.D.
PUBLISHED BY JAMES P.HARRISON&.CGK
__________________Atlanta ,ga.
SUBSCRIPTION : 2.30 A YEAR.
				

